# Social Interaction Patterns of the Disabled People in Asymmetric Social Dilemmas

**DOI:** 10.3389/fpsyg.2018.01683

**Published:** 2018-09-19

**Authors:** Shen Liu, Wenlan Xie, Shangfeng Han, Zhongchen Mou, Xiaochu Zhang, Lin Zhang

**Affiliations:** ^1^Department of Psychology and Institute of Psychology, Ningbo University, Ningbo, China; ^2^School of Humanities and Social Science, University of Science and Technology of China, Hefei, China; ^3^Ningbo Institute of Education, Ningbo, China; ^4^School of Psychology, Nanjing Normal University, Nanjing, China; ^5^Hefei Medical Research Center on Alcohol Addiction, Anhui Mental Health Center, Hefei, China; ^6^CAS Key Laboratory of Brain Function and Disease, and School of Life Sciences, University of Science and Technology of China, Hefei, China; ^7^Academy of Psychology and Behavior, Tianjin Normal University, Tianjin, China

**Keywords:** social interaction, social dilemmas, asymmetry, cooperation, the disabled people

## Abstract

The social participation of the disabled people is unsatisfactory and low, one of the reasons often overlooked but of great importance may lie in the disparate patterns of social interaction between the disabled people and the abled people. The current study respectively recruited 41 and 80 disabled people in two experiments and adopted give-some games and public good dilemma to explore social interaction patterns between the disabled abled people. The results were as follows: (1) the disabled people preferred to interact with the disabled people and the abled people preferred to interact with the abled people. (2) The disabled abled people had higher cooperation, satisfaction and sense of justice when interacting with the disabled people than interacting with the abled people. (3) Advantage in the number of the disabled people could reverse their disadvantage in the identity. These results are of important practical value, which provides related theoretical support for the disabled people’s federation and communities when carrying out activities for the disabled people.

## Introduction

With the continuous improvement of social security for the disabled people, the objective conditions such as income and living conditions of the disabled people have improved remarkably while the quality of social life of the disabled people has not. In particular, the social participation of the disabled people is still unsatisfactory and low (Zhang et al., 2014). The reasons for the current social participation of the disabled people are various, such as their limited physical abilities (Lu et al., 2017), perceived discrimination resulting from physical disabilities (Zhang et al., 2014). However, one of the reasons often overlooked but of great importance may lie in the disparate patterns of social interaction between the disabled and abled people. Because of their own and social reasons, the disabled people are at a disadvantage in their social status and resource distribution. Therefore, they are in an unequal position in their interactions with abled people, which results in their low level of social participation.

The social interactions of the disabled people are not optimistic with simple social network, simple social interactive object and low social interaction willingness as the main manifestations of their difficulties in social interactions. Reasons for the disabled people’s difficulties in social interactions may lie in the following aspects: (1) one is that the abled people tend to show negative attitudes (e.g., social stigma) and behaviors to the disabled people in daily lives (Zhang et al., 2015). For example, it is reported that the disabled people claimed discriminations from their peers (Moore et al., 2011) and families (O’Reilly et al., 2016). The abled people have an inherent prejudice against the disabled people that results in the formation of public stigma (Forber-Pratt et al., 2017). While on the other hand, the disabled people internalize the public stigma. They recognize and accept the cultural stereotype of the group they live in and apply it to themselves, and thus self-stigma forms (Ditchman et al., 2013); (2) the other possible reason is the unequal status in social interactions between the disabled and abled people. The disabled are on the fringes of society, both in terms of accessing to social resources and the distribution of living environment as well as working opportunities, socio-economic status and quality of life, compared with abled people (Riddell and Weedon, 2014). Such an unequal status may be the core reason why the disabled people do not want to participate in social interactions and establish good relationships with other people, especially the abled people. However, the studies aiming directly at the social interaction patterns between the disabled and abled people in unequal status are still rare.

Cooperation and competition are basic forms of social interactions. People need to have social interactions and exchanges of resources with others for survival and development in society (Wang et al., 2016). Cooperative behavior is an important factor in maintaining good social interactions as well as the redistribution of interests of social subjects. Therefore, cooperative behavior is of particular importance for the disabled people who are at a disadvantage of resource distribution and social status. For example, a possible reason why the disabled people show low cooperation is that they may take into account their own economic conditions or status when interacting with the abled people in the hope of making up for these deficiencies in the distribution of resources so as to distribute more resources to themselves and less resources to the abled people. Even worse, the disabled people’s low cooperation and unfriendliness may in turn affect the abled people’s attitudes and behaviors toward them, such as indifference or avoidance (unwillingness to interact with the disabled people). In summary, the different patterns of social interactions between the disabled and abled people may be the important reasons for the difficulties the disabled people encounter when integrating into the society. In addition, the disabled people’s disadvantaged status caused by physical or mental defects are unlikely to change in a short period and are irreversible (Brown and Brunell, 2017). Based on the theories of cooperation and competition under unequal status and social dilemmas paradigms in the field of decision making, the current study intended to explore social interaction patterns between the disabled and abled people under the conditions of unequal resources and status.

Social dilemma is a situation in which the interests of an individual and groups conflict. The benefits of members choosing not to cooperate in this situation are higher than those of choosing cooperation, but the overall benefit of all members choosing to cooperate is greater than the benefit of defection (Roch and Samuelson, 1997; Wang and Chen, 2011; Liu and Hao, 2014). Based on the hypothesis of “rational man,” the classical game theory holds that the two parties of a game will make their own decisions in accordance with the maximization of their own interests in a dilemma situation. However, in many social dilemmas, the two parties of a game still choose to cooperate which seems irrational and the distribution tend to be fair (Roth, 1991; Martínez-Cánovas et al., 2016). In addition, individuals are constrained by their own resources and interactive situations when weighing their own and others’ interests. In social dilemma models, researchers often assume that participants have equal resources or status prior to distribution. However, in real lives, the two competing parties often have strengths and weaknesses due to a variety of reasons, which in turn will lead to different levels of dominance. When it is projected into social interactions, asymmetric social dilemmas are formed (Liu and Hao, 2015). In asymmetric social dilemmas, the existence of dominance gives part of the population more opportunities than others to access resources that contribute to the survival and reproduction of them. It is also for this reason that the dominance level induces inevitable conflicts between the dominating and the dominated individuals. For example, inequalities in resources, income, power and the like can hinder cooperation from taking place (Liu and Hao, 2014; Hao et al., 2016). However, some studies showed that a certain degree of inequality had a positive effect on cooperative behaviors, namely the theory of “disadvantage makes people more cooperative” (Zitek and Tiedens, 2012; He et al., 2014). Therefore, the effect of unequal status on cooperative behaviors is very complicated. However, the social dilemmas confronted by the disabled people in the current study were different from those by general population. The disadvantaged situation of the disabled people caused by physical or psychological defects was irreversible. However, in the previous studies, disadvantaged status resulting from inequality in resources, interests and power could be somewhat altered. Therefore, the effect of the irreversible inequalities on the cooperative behaviors of the disabled people may be more complex than that of the general population. Hence, exploring the patterns of social interaction between the disabled and abled people can not only help us to understand the difficulties the disabled people encounter in social interactions but also enhance the social participation of them. On the other hand, it can help us to understand the characteristics of social interactions between vulnerable groups represented by the disabled people and advantaged groups represented by the abled people.

There are many theories explaining cooperation under unequal status. For example, Trivers put forward the reciprocity theory and thought that the essence of cooperative behaviors was the exchange of interests among individuals, namely they can choose either to “cooperate” or “defect” (Trivers, 1971). Reciprocity is divided into strong and weak reciprocity, while weak reciprocity is manifested as direct reciprocity and indirect reciprocity (Tsvetkova and Buskens, 2013). Direct reciprocity occurs between two persons and its principle is “you help me and I will help you” (Sigmund, 2012). The indirect reciprocity is to gain mutual benefits from others through reputation and its principle is “you help me and others will help you” (Sigmund, 2012). However, neither direct nor indirect reciprocity can explain individuals’ cooperative behaviors when they face threats such as war, plague or famine that threaten the survival of the community (Rao et al., 2011). In these situations, as the probability of group disintegration increases, the probability of survival of the entire population declines. As a result, time was extremely valuable and those who could not wait for the third parties’ reward often chose to defect. However, once the defection spreads among the group, the destruction of it will soon follow. Therefore, in order to ensure that the group will not disintegrate, the members of the group will punish the betrayals at the expense of their own interests, which is called “strong reciprocity.” The existence of strong reciprocity individuals ensures more benefits of the group than the price paid by the individuals. What’s more, as the group is also more inclined to favor those who are willing to bear the costs and protect the interests of the masses, they are more likely to survive in the group and thus strong reciprocity evolves. Rao et al. (2011) put forward the theory of “disadvantage makes people more cooperative” and thought there was a set of system in human genes to improve the probability of reproduction and survival of individuals. Disadvantaged individuals need to increase their chances of survival and reproduction through cooperation due to their weak competiveness. Compared with the reciprocity theory, this theory can explain the mechanism of pro-social behaviors more succinctly and effectively. While the “fairness theory” is a competing assumption that indicates individuals have a perception of justice or averseness to injustice in making decisions (Xiao, 2013). During the game, both parties of the interaction will have “consensus” on “recognition of justice.” In other words, they tend to reduce the unfairness during the distribution process in cooperative decision-making (Vandello et al., 2011). Hence, the reciprocity theory, the theory of “disadvantage makes people more cooperative” and the fairness theory all could explain cooperative behaviors under unequal situations to some extent. However, as a special group, the asymmetric situations formed by the interactions of the disabled people with the abled are different from those formed by laboratory manipulation. Therefore, it is also one of the issues the current study tended to explore that whether the interaction patterns between the disabled and abled people followed the above theories or assumptions.

Social dilemmas often involve two or more people (Liu and Hao, 2014). According to the number of people involved, social dilemma can be divided into two-person dilemma and multiple-person dilemma (Rand, 2017). The social interactions of individuals are not just one-to-one interactions, but interactions involving different groups, such as the disabled/abled people may interact with the abled/disabled people or mixed group (including the disabled and abled). Two-person interaction is the simplest social relationship. Although it also has the characteristics of social dilemma, social dilemma is often manifested as multiple-person social interactions (Liu and Hao, 2014). Individuals’ psychological and behavioral performances in a two-person dilemma are different from those of multiple-person dilemma (EL-Seidy et al., 2016; Płatkowski, 2016). Therefore, it is necessary to study the social interaction patterns between the disabled people and the abled people in two-person and multiple-person interactions. The current study doubted whether there were changes in psychological feelings caused by the changes in the number of the disabled and abled people in multiple-person interactions. Another question the current study intents to answer is that whether there is any difference in social interaction patterns between the disabled and the abled people.

The current study carried out two experiments to investigate two-person and multiple-person social interaction patterns by using give-some games and public good dilemma. According to the previous studies (Radke et al., 2014; Gilam et al., 2015), two indexes are used to investigate individuals’ social interaction behaviors and results. One is objective index, namely cooperative behaviors (distribution of resources in social dilemmas). The other is subjective index, which includes initial social interaction tendencies and psychological feelings during interaction processes. In general, the current study tried to answer four questions. Firstly, which group (the disabled people or the abled people) does the disabled people prefer to interact with? Secondly, does the disabled people have the same social interaction patterns (including cooperation and psychological feelings) as that of the abled people? Thirdly, is the social interaction patterns of the disabled people influenced by the change in status in multiple-person interactions? Fourthly, which theory of asymmetric game could better explain social interaction patterns between the disabled people and the abled people? Based on these, the current study put forward the following hypotheses. Hypothesis one: The disabled people will prefer to interact with the disabled people and the abled people will prefer to interact with the abled people. Hypothesis two: The disabled people will have higher cooperation when interacting with the disabled people than with the abled people, the abled people will have higher cooperation when interacting with the disabled people than with the abled people. Hypothesis three: The disabled people will have higher satisfaction and sense of justice when interacting with the disabled people than with the abled people. Hypothesis four: Asymmetric status will affect the disabled and abled people’s cooperation and the disabled people can use their superiority in number to make up their inferiority in the status.

The current study aimed to reveal the disabled people’s strategies of selection in the face of conflicts between personal interests and others’ interests under social dilemmas to further explore human nature and to promote altruistic behaviors of human beings and social development.

## Experiment 1

### Participants

The current experiment randomly recruited 41 disabled people including 23 males and eighteen females with an average age of 51.65 years old (*SD* = 10.55) and forty abled people were randomly recruited including twenty-two males and eighteen females with an average age of 50.25 years old (*SD* = 10.59). All disabled participants met the national standard for disabled people and they were mostly Grade II or Grade III of physical disability (mainly as disabilities in arms of legs) with the mean time of disability for 23.6 years. All participants had normal or corrected-to-normal vision, with no partial tritanopia or achromatopsia, and could skillfully operate the computer. The present study was approved by the Ethics Committee of the authors’ University in accordance with the ethical principles of the Declaration of Helsinki. All subjects gave written informed consent in accordance with the ethical principles of the Declaration of Helsinki.

### Materials

#### Social Interaction Tendency

The measurement of social interaction tendency was based on previous studies (Radke et al., 2014; Gilam et al., 2015). We respectively adopted a question to measure social interaction tendency including “Which group do you prefer to contact/communicate with in daily life? 1 = Disabled people, 2 = Abled people.”

#### Cooperation

The measurement of cooperation level was based on Liu and Hao (2014). Repetitive give-some games were adopted to set up the social dilemmas of two-person interactive situations. Before the experiment, participants owned certain amount of initial monetary resource, which was 100 RMB. Then, participants could distribute initial monetary resource to the others. The amount of the distribution represented the participants’ cooperation level. Subsequently, participants distributed initial monetary resource according to the instructions (see details in the **Supplementary Information**).

#### Psychological Feelings

The measurement of psychological feelings (satisfaction and justice) during social interactions was based on previous studies (Radke et al., 2014; Gilam et al., 2015). For satisfaction, the question was “Are you satisfied with the previous round of interaction, including the performance of the other and yours and overall experience?” For sense of justice, the question was “do you think the amount your partner distributed to you is fair during the 10 rounds of distribution?” The two questions are all rated with five-point scale ranging from 1 (extremely unsatisfied or extremely unjustified) to 5 (extremely satisfied or justified).

### Experimental Design

A two factor between-subject design with types of participants and interactive objects both including two levels as the disabled people and the abled people was adopted. Dependent variables included cooperation and psychological feelings. The cooperation referred to the amount of money the participants distributed to the other person (experiment one) or the public (experiment two) and psychological feelings referred to participants’ satisfaction and sense of justice during social interactions.

### Task and Procedure

All the materials were presented on the computer screen, and participants were ordered to conduct 10 transactions with the interactive objects randomly selected via the computer. In addition, all interactive objects were virtual. Computers were connected to the Internet and participants could obtain information they needed at any time. In each round of transaction, participants and interactive objects each owned gifts worth 0–100 RMB. They had to offer to each other corresponding gifts. When each interaction was finished, they all received gifts offered by virtual interactive objects. The current experiment let virtual interactive objects imitate actual interactive objects’ general distribution. Individuals often tend to offer resource equally (Martínez-Cánovas et al., 2016). Therefore, the mean of feedback of the ten rounds were 51.4 and five rounds were higher than 50 and five rounds were lower than 50. This number came from ten numbers selected randomly, which was pseudo-random. In other words, feedback-based numbers were randomly selected as higher and lower than 50. Participants’ cooperation in each trial could be compared by a fixed sequence order. The participants were all notified that the total value they would receive after ten-round investment was the true value of gifts after the experiment (but in fact the true value was a fixed amount irreverent to the amount of the experiment). Only when the participants correctly answered and offered the money could they enter the formal experiment. Before the experiment, social interaction tendency of the disabled and abled people needed to be measured and satisfaction and sense of justice were also needed to be rated after every round.

### Results

#### Social Interaction Tendency

Among 41 disabled people, 56.1% of them preferred to interact with the disabled people. Among 40 abled people, 97.5% of them preferred to interact with abled people. A Chi-square test found that the disabled people preferred to interact with the disabled people [χ^2^(12) = 36.41, *p* = 0.052] and abled people preferred to interact with abled people [χ^2^(15) = 58.99, *p* < 0.01].

#### Cooperation

**FIGURE 1:**
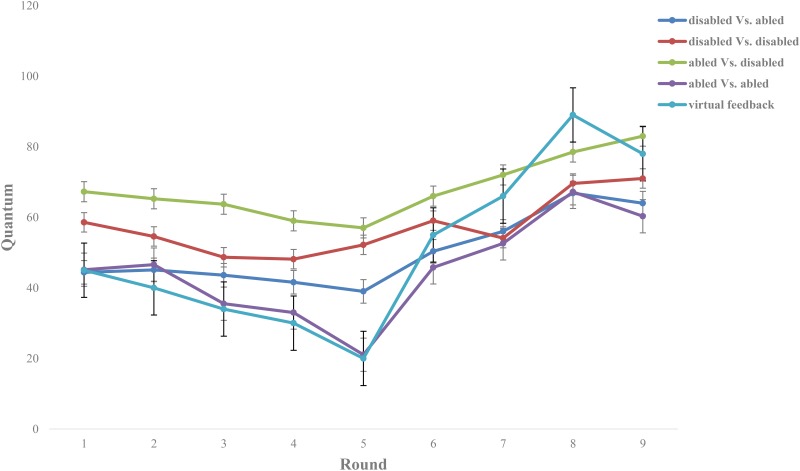
Feedback and different participants’ cooperation in each round during ten rounds. The amount given in the interaction between the disabled people and the disabled people was significantly higher than the fair baseline (*p* < 0.05) while there was no significant difference between the amount given in the interaction between the disabled people and the abled people and the fair baseline (*p* = 0.645). Moreover, the investment given in the interaction between the abled people and the disabled people was significantly higher than the fair baseline (*p* < 0.001) while the investment given in the interaction between the abled people and the abled people was significantly lower than the fair baseline (*p* < 0.001). It indicated that there was a high level of cooperation in the interaction between the disabled people and the disabled people while the disabled people tended to distribute equally when interacting with the abled people. Moreover, there was a high level of cooperation in the interaction between the abled people and the disabled people while the disabled people showed comparatively rational selfishness when interacting with the abled people.

In experiment one, the main effect of the types of participants was not significantly different [*F*_(1,77)_ = 0.40, *p* = 0.530] indicating that there was no difference in the amount given to the peers between the two different types of participants. There was a significant difference of the main effect of interactive objects [*F*_(1,77)_ = 6.65, *p* < 0.05, $\eta_{p}^{2}$ = 0.08] indicating that there was a significant difference in the investment given during the interaction. The cooperation during the interaction between the disabled people and the disabled people (*M* = 58.72) was significantly higher than that between the disabled people and the abled people (*M* = 49.87; *p* < 0.01) while the cooperation during the interaction between the abled people and the disabled people (*M* = 68.15) was significantly higher than that between the abled people and the abled people (*M* = 44.15; *p* < 0.001). Taking the average feedback value (*M* = 51.4) from the 10-round interactions with the virtual interactive object as the fair baseline, we analyzed whether the investment of the disabled people and the abled people would be higher or lower than the fair baseline (see **Figure 1**).

#### Psychological Feelings

In experiment one, for the results of satisfaction, there was no significant main effect of the types of participants [*F*_(1,77)_ = 1.15, *p* = 0.704] indicating that there was no difference of satisfaction of social interactions for the two groups. There was a significant main effect of interactive objects [*F*_(1,77)_ = 24.66, *p* < 0.001, $\eta_{p}^{2}$ = 0.24] indicating that there was a significant difference in participants’ satisfaction with different interactive objects. Moreover, the interaction was significant [*F*_(1,77)_ = 24.66, *p* < 0.001, $\eta_{p}^{2}$ = 0.24]. Satisfaction in the interaction between the disabled people and the disabled people (*M* = 4.33) was significantly higher than that between the disabled people and the abled people (*M* = 2.90; *p* < 0.001) while there was no difference in satisfaction in the interaction between the abled people and the disabled people (*M* = 3.4) and the abled people and the abled people (*M* = 3.7; *p* = 0.203). For the results of justice, there was a significant main effect of types of participants [*F*_(1,__77)_ = 4.59, *p* < 0.05, $\eta_{p}^{2}$ = 0.06] and the disabled people’s justice perception (*M* = 3.66, *SD* = 0.88) was higher than that of the abled people (*M* = 3.25, *SD* = 0.84). There was a significant main effect of interactive objects [*F*_(1,77)_ = 4.59, *p* < 0.05, $\eta_{p}^{2}$ = 0.06] and the cooperation of the same group (*M* = 3.66, *SD* = 0.73) was significantly higher than that of different groups (*M* = 3.25, *SD* = 0.98). Moreover, the interaction was not significant [*F*_(1,77)_ = 1.16, *p* = 0.286].

## Experiment 2

### Participants

The current experiment randomly recruited eighty disabled people including 44 males and 36 females whose mean age was 52.67 years (*SD* = 9.38) and eighty abled people including forty-one males and thirty-nine females whose mean age was 48.21 (*SD* = 9.47). All disabled participants met the national standard for the disabled people and they were mostly Grade II or Grade III of physical disability (mainly as disabilities in arms of legs) with the mean time of disability for 22.8 years. All participants had normal or corrected-to-normal vision, with no partial tritanopia or achromatopsia, and could skillfully operate the computer. The present study was approved by the Ethics Committee of the authors’ University in accordance with the ethical principles of the Declaration of Helsinki. All subjects gave written informed consent in accordance with the ethical principles of the Declaration of Helsinki.

### Materials

#### Social Interaction Tendency

The measurement of social interaction tendency was based on previous studies (Radke et al., 2014; Gilam et al., 2015). We respectively adopted a question to measure social interaction tendency including “Which group do you prefer to contact in daily life? (A) Three disabled people; (B) Two disabled people and one abled people; (C) One disabled people and two abled people; (D) Three abled people.” Participates sort the order according to the range from the most willingly to participate to the most unwillingly to participate with the highest ranked as four points and the lowest ranked as one point” to show their social interaction tendency.

#### Cooperation

The measurement of cooperation level was based on Liu and Hao (2014). Public good dilemma was adopted to set up social dilemmas of multiple-person interactions of the current experiment. It was assumed that four people completed a decision task together and each one had a personal and a group account. The personal account was only used by participants and the group account was used by all members of the group. Everyone needed to distribute their initial resource to the personal and group account and the amount distributed to the group account stood for the cooperation level of participants (see details in the **Supplementary Information**).

#### Psychological Feelings

The measurement of psychological feelings (satisfaction and justice) during social interactions in experiment two was the same as in experiment one.

### Experimental Design

A two factor between-subject design with types of participants and interactive situations was adopted. Types of participants included two levels: the disabled people and the abled people, and the interactive situations included four levels: the single identity group, the advantage group, the peer group and the disadvantage group. The constitutions of these four groups were shown in **Table 1**. Except the single identity group, the other three groups were all mixed group, which contained both the disabled and abled people. Dependent variables in experiment two were the same as in experiment one.

**Table 1:** Four groups of the interactive situations in experiment two.

Interactive situations	Constitutions
The single identity group	(I) One disabled people interacting with three virtual disabled people
	(II) One abled people interacting with three virtual abled people
The advantage group	(I) One disabled people interacting with two virtual disabled people and one virtual abled people
	(II) One abled people interacting with two virtual abled people and one virtual disabled people
The peer group	(I) One disabled people interacting with one disabled people and two abled virtual people
	(II) One abled people interacting with one abled people and two virtual disabled people
The disadvantage group	(I) One disabled people interacting with three virtual abled people
	(II) one abled people interacting with three virtual three disabled people


### Task and Procedure

Participants needed to complete a ten-round investment task on the computer with other randomly selected (virtual) participants. Everyone had a personal account and the group had a public account. The personal account belonged to the participants and contained an initial amount of 100 RMB. Participants could distribute any amount of the personal account (0–100 RMB) to the public group. When the amount of the public account reached or exceeded 200 RMB, the amount of the public account would double and was averagely distributed to the group members. Whether investment or not, the investment would be confiscated when the amount of the public account did not reach 200 RMB. The total value after 10-round investment was the true value of the gifts after the experiment. When participants read instructions, they needed to complete some task-related computations. Then, they needed to complete five practicing trials to familiar themselves with the experiment. Only when participants correctly answered and distributed the money could they enter the formal experiment. The disabled and abled people were randomly assigned to different multiple-person interactive situations.

### Results

#### Social Interaction Tendency

There was a significant difference in the preference of the disabled people for the different types of interactive situations [*F*_(3,237)_ = 5.36, *p* < 0.001, $\eta_{p}^{2}$ = 0.06] and the range from high to low was the single identity group (*M* = 2.86, *SD* = 1.05), the peer group (*M* = 2.58, *SD* = 0.87), the advantage group (*M* = 2.49, *SD* = 1.04) and the disadvantage group (*M* = 2.08, *SD* = 1.34). The planned *t*-test found that the social interaction tendency of the disadvantage group was significantly lower than that of the single identity group (*p* < 0.001) and the peer group (*p* < 0.05), which indicated that the disabled people preferred to interact with the same type of individuals and preferred not to interact with the disadvantage group in the multiple-person interactive situations. In addition, there was a significant difference in the preference of the abled people for different types of interactive situations (*F*_(3,237)_ = 34.45, *p* < 0.001, $\eta_{p}^{2}$ = 0.30) and the range from high to low was the single identity group (*M* = 3.48, *SD* = 1.03), the advantage group (*M* = 2.48, *SD* = 0.95), the peer group (*M* = 2.15, *SD* = 0.83) and the disadvantage group (*M* = 1.85, *SD* = 0.92). The planned *t*-test found that there was a significant difference among each interactive situations (*ps* < 0.05), which indicated that the abled people preferred to interact with the abled people and the preference tended to decrease with the decrease in the proportion of the abled people in the group.

#### Cooperation

**FIGURE 2:**
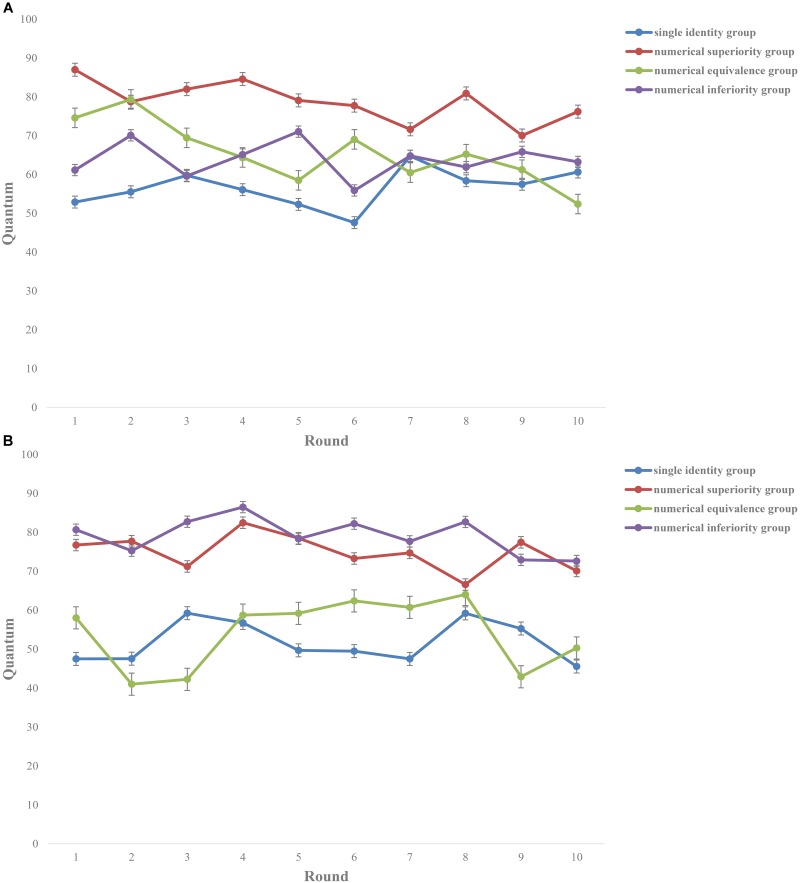
Cooperation of **(A)** the disabled people and **(B)** the abled people in ten rounds, respectively. The results showed that the amount the disabled people invested in the single identity group, in the advantage group, in the peer group and in the disadvantage group were all higher than the fair baseline (*ps* < 0.05). There was no difference of the amount the abled people invested in the single identity group (*p* = 0.175) and in the peer group (*p* = 0.079) compared with the fair baseline while the investment in the advantage group and in the disadvantage group was significantly higher than the fair baseline (*ps* < 0.05). It indicated that the advantage group and the disadvantage group highlighted the “individuals’ unequal status,” which resulted in their higher level of cooperation than the fair baseline.

There was no significant main effect of the types of the participants [*F*_(1,152)_ = 0.24, *p* = 0.622] indicating that there was no difference in the public goods investment in the multiple-person interactions between the two groups. There was a significant main effect of interactive situations [*F*_(1,152)_ = 24.64, *p* < 0.001, $\eta_{p}^{2}$ = 0.33] indicating that there was a significant difference in the public goods investment in different interactive situations. Moreover, the interaction was significant [*F*_(1,152)_ = 7.63, *p* < 0.001, $\eta_{p}^{2}$ = 0.13]. In addition, we set 50 as the fair baseline to analyze the investment of the disabled people and the abled people (see **Figure 2**).

#### Psychological Feelings

For the satisfaction, there was no significant main effect of the types of participants [*F*_(1,152)_ = 0.012, *p* = 0.911] indicating that there was no difference of satisfaction of social interactions for the two groups. There was a significant main effect of interactive situations [*F*_(1,152)_ = 11.42, *p* < 0.001, $\eta_{p}^{2}$ = 0.18] indicating that there was a significant difference in participants’ satisfaction in different interactive situations. Moreover, the interaction was significant [*F*_(1,152)_ = 9.26, *p* < 0.001, $\eta_{p}^{2}$ = 0.16]. For the justice, there was no significant main effect of types of participants [*F*_(1,152)_ = 1.62, *p* = 0.205] indicating that there was no difference in justice of social interactions for the two groups. There was a significant main effect of interactive situations [*F*_(1,152)_ = 3.20, *p* < 0.05, $\eta_{p}^{2}$ = 0.06] indicating that there was a significant difference in participants’ justice in different interactive situations. Moreover, the interaction was significant [*F*_(1,152)_ = 8.49, *p* < 0.001, $\eta_{p}^{2}$ = 0.14].

## Discussion

The current experiment revealed possible social interaction patterns of the disabled abled people in social interactions. The disabled people preferred to interact with the disabled people and the abled people preferred to interact with the abled people, which was consistent with the previous studies (Zeedyk et al., 2014). These results confirmed the hypothesis one. This asymmetry might indicate that disabled people’s preference for interactive objects was lower than the abled people. In the social interactions, the abled people might make a more explicit distinction between these two groups compared with the disabled people. The disabled people had a higher cooperation when interacting with the disabled people than interacting with the abled people and the abled people had higher cooperation when interacting with the disabled people than interacting with the abled people. These results confirmed the hypothesis two. The interaction between the disabled abled people appears to be more compliant with the fairness theory. In other words, the cooperation level during the interactions between the disabled abled people was lower than that between the abled disabled people. It indicated that the disabled people at a disadvantage were more sensitive to the equality of the distribution (Zeng et al., 2016) for there was no significant difference between the distribution of the disabled abled people and the fair baseline. It also indicated that the abled people’s “unequal averseness” made them tend to narrow the gap in the distribution to distribute more to the opposite, namely the high distribution of the abled people could be perceived by the disabled as unreasonable respect. During the whole interactions, although there were differences in participants’ average distribution, participants’ cooperation levels in every round were all influenced by the opposite. It might indicate that no matter the disabled abled people, their social behaviors and social attitudes were all influenced by interactive objects in daily lives.

The current experiment also found that the change in the member of groups did affect the cooperation between the disabled abled people. For the disabled people, although they preferred to interact with their own groups and the peer group, their cooperation level in the single identity group and in the peer group was low while their cooperation level in the advantage group was high. These results confirmed the hypothesis four. Previous studies found that the disabled people entering the integrated environment, which is comprised of the disabled people, can promote their participation and interactions in physical activities (Bossaert et al., 2013). However, other studies found that the integrated environment may restrict some psychological factors and put forward the reverse integration (RI) environment. It was thought that the disabled people under this environment had lower desire to integrate (Rao et al., 2011). The current experiment also confirmed that the RI environment could relieve the disabled people’s psychological disadvantage in asymmetric status to some extent. Moreover, the cooperation level declined as the number of disabled people decreased in multiple-person interactions. These results supported the justice theory and clarified that “disadvantage makes people more cooperative” might only be feasible in the two-person interaction and in the peer group. When the disabled people’s advantage in the number reversed their disadvantage in the status, social interaction patterns of the disabled people were the same as those of the abled people. For the abled people, the cooperation level was higher in the advantage group and in the disadvantage group. The former was that the abled people were in advantage in number and status and they needed high devotion to narrow the gap, which supported the justice theory. The latter might be that one single abled people in the group would highlight his advantage in status or might be that disadvantage in number stimulated higher cooperation. However, the current study could not interpret individuals’ inclination of decision when their identity and number were at disadvantage simultaneously. In addition, there was higher level of cooperation of the disabled people in two-person and multiple-person interactions of the single identity group and the peer group compared with the abled people, which was consistent with the results of the two-person interactions and further supported “disadvantage makes people more cooperative.”

As for the psychological feelings, the current experiment found that although interactive objects’ feedback was the same, the disabled people had a higher level of satisfaction and sense of justice when interacting with the disabled people. These results confirmed the hypothesis three. It explains to some extent the reason why the disabled people do not want to interact with the abled people. In the meanwhile, the abled people’s high distribution did not improve the disabled people’s satisfaction, which might be related to the fact that the disabled people did not regard the high distribution as respect. Chen and Shu (2012) also found that the disabled students’ disabled identity could on the one hand gives them extra help, but on the other hand be regarded as one of the source of stigma. In daily lives, the disabled people may have misunderstanding and prejudice against the abled people so that they don’t want or evade interacting with the abled people.

Based on the social game theory and its paradigms, the current study explored social interaction patterns of the disabled people in asymmetric dilemmas and has some significant meanings. Firstly, exploring social interaction patterns between the disabled people and the abled people in unequal situations of resource and status is conducive to deepening the publics’ understanding of the disabled people’s social interaction patterns and feelings, and encouraging more the disabled people to participate social interaction. Secondly, it is both of great theoretical and practical significance to understand the social interaction dilemmas of the disabled people, to improve social participation of the disabled people, to strengthen publics’ understanding of the disabled people’s social behaviors, and to deepen and extend researches of vulnerable groups. It also enhances the awareness of the disabled people about their and other people’s behaviors, improves their cognition of self-stigma and social interaction. Thirdly, it provides theoretical and practical evidence for the government, the community and other organization to establish policies or hold activities. Fourthly, it will help the relevant departments of the government, community service organizations for the disabled and other relevant organizations to formulate policies and regulations or carry out activities that are beneficial to the physical and mental health of the disabled people, as well as to provide theoretical and empirical evidence for caring for and interacting with the disable people effectively, scientifically and rationally.

However, there are some limitations of the current study that need to be improved in the future studies. Firstly, the participants of the current study were special groups and experimental procedure was comparatively complex. Therefore, the sample size may be small and not representative enough. In particular, the sample collection was mainly concentrated in urban areas, the lack of samples in other areas such as rural areas, may affect the generalization of the findings. If conditions permit, a larger sample size and expanded sample collection area will be required in future studies. Secondly, the current study not only focused on intragroup cooperation of the disabled people, but also on intergroup cooperation between the disabled people and the abled people, which expanded researches of cooperation in asymmetric social dilemmas. However, the current study adopted simplified real-life dilemmas, which reflected abstract social dilemmas. Although the simplified real-life dilemmas in the current study also included some real-life factors (e.g., multiple interactions, feedbacks), the behavioral index was too simple. Other behavioral variables need to be combined in future studies in order to carry out more comprehensive researches. Thirdly, the psychological indexes in the current study did not correspond well to the behavioral indexes due to the measurement of only using a single or twofold items. Therefore, the psychological indicators on cooperative behaviors of the disabled still need to be improved in future researches.

## Ethics Statement

The current study was implemented in conformity to the recommendations of the Ethical Committee of Ningbo University. Informed consent of all participants was obtained in line with the Declaration of Helsinki. The protocol was approved by the Ethical Committee of Ningbo University.

## Author Contributions

LZ and XZ designed and implemented the study. SL, WX, SH, and ZM analyzed the data. SL, LZ, and XZ interpreted the data. SL, WX, SH, ZM, LZ, and XZ wrote the manuscript.

## Conflict of Interest Statement

The authors declare that the research was conducted in the absence of any commercial or financial relationships that could be construed as a potential conflict of interest.
