# Supplementary material for: Social Interaction Patterns of the Disabled People in Asymmetric Social Dilemmas
Source: Front Psychol. 2018 Sep 19;9:1683. doi: 10.3389/fpsyg.2018.01683 (PMC6157442; doi:10.3389/fpsyg.2018.01683)
Supplement: Supplementary file 1 [file Table_1.DOC]

**Supplementary Information**

**S1 Give-some games**

Now you have some gifts worth 0-100 RMB. You and your partners will exchange gifts in ten rounds. During the exchange, you’ll need to fill in the amount of money you want to exchange. Then the following question will appear on the screen:


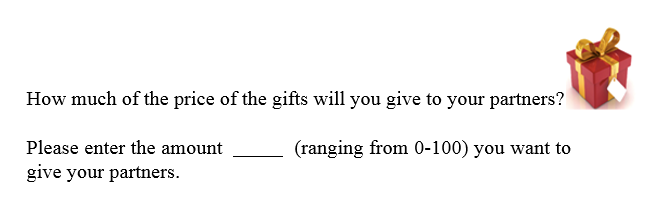


When participants enter the amount of money and press the spacebar, the computer will give the following feedback.


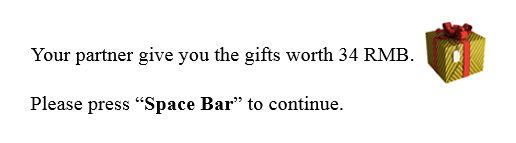


This is a single round and participants need to complete ten rounds.

**S2 Public good dilemma**

You and other three people co-invested a hotel and each one invests 100 RMB. When the investment of four people exceeds or equals to 200 RMB, the hotel will be opened. In addition, the turnover will be the double of the total investment and the profit will be divided equally to the four people. If the total investment is less than 200 RMB, the hotel cannot be opened.

To ensure that the participants understand the instruction, two specific examples are presented to the participants before the formal experiment begins.

If other three people invest a total of 150 RMB and you invest 50 RMB, the total investment is 150 plus 50 equals 200 RMB. Now that the total investment exceeds or equals to 200 RMB, you and the other three people will make a profit. Accordingly, the amount you will receive is 200 times 2 divided by 4 equals 100 RMB. However, if other three people co-invest 120 RMB and you invest 50 RMB, the total amount is 120 plus 50 equals 170 RMB which is below 200 RMB, and you will not make a profit.

Besides, participants are also required to calculate. If the other three people co-invest 140 RMB and you invest 80 RMB, the total amount is 140 plus 80 equals 220 RMB. Can you make a profit and how much? Participants are then randomly assigned to four situations. For example:


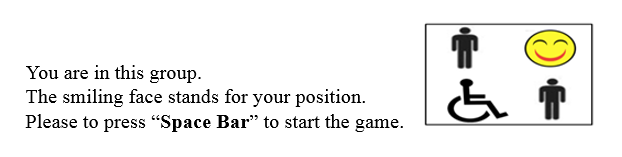


Then the question will appear.


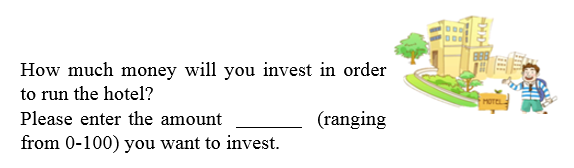


When participants enter the amount of money and press the spacebar, the computer will give the following success or failure feedback.


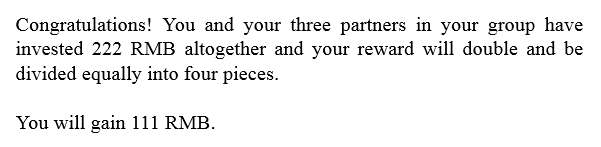


This is a single round and participants need to complete ten rounds.
